# Body Temperatures in Dinosaurs: What Can Growth Curves Tell Us?

**DOI:** 10.1371/journal.pone.0074317

**Published:** 2013-10-30

**Authors:** Eva Maria Griebeler

**Affiliations:** Department of Ecology, Zoological Institute, University of Mainz, Mainz, Germany; Raymond M. Alf Museum of Paleontology, United States of America

## Abstract

To estimate the body temperature (BT) of seven dinosaurs Gillooly, Alleen, and Charnov (2006) used an equation that predicts BT from the body mass and maximum growth rate (MGR) with the latter preserved in ontogenetic growth trajectories (BT-equation). The results of these authors evidence inertial homeothermy in Dinosauria and suggest that, due to overheating, the maximum body size in Dinosauria was ultimately limited by BT. In this paper, I revisit this hypothesis of Gillooly, Alleen, and Charnov (2006). I first studied whether BTs derived from the BT-equation of today’s crocodiles, birds and mammals are consistent with core temperatures of animals. Second, I applied the BT-equation to a larger number of dinosaurs than Gillooly, Alleen, and Charnov (2006) did. In particular, I estimated BT of *Archaeopteryx* (from two MGRs), ornithischians (two), theropods (three), prosauropods (three), and sauropods (nine). For extant species, the BT value estimated from the BT-equation was a poor estimate of an animal’s core temperature. For birds, BT was always strongly overestimated and for crocodiles underestimated; for mammals the accuracy of BT was moderate. I argue that taxon-specific differences in the scaling of MGR (intercept and exponent of the regression line, log-log-transformed) and in the parameterization of the Arrhenius model both used in the BT-equation as well as ecological and evolutionary adaptations of species cause these inaccuracies. Irrespective of the found inaccuracy of BTs estimated from the BT-equation and contrary to the results of Gillooly, Alleen, and Charnov (2006) I found no increase in BT with increasing body mass across all dinosaurs (Sauropodomorpha, Sauropoda) studied. This observation questions that, due to overheating, the maximum size in Dinosauria was ultimately limited by BT. However, the general high inaccuracy of dinosaurian BTs derived from the BT-equation makes a reliable test of whether body size in dinosaurs was ultimately limited by overheating impossible.

## Introduction

The thermal physiology of dinosaurs has long been a topic of interest and is still intensively discussed [1-7]. The debate mainly focuses on the question whether dinosaurs were endotherms or ectotherms [3]. As in extant species, the process of thermoregulation is very complex; this endotherm/ectotherm dichotomy seems to be too simplistic [3,8].

Endotherms, such as today’s mammals and birds make use of an internal heat source. They show high body temperatures that are relatively constant. The rather constant core temperature of endothermic animals comes at a metabolic cost [9-11], which is particularly significant in very small individuals [12] and in those living in environments with temperatures strongly deviating from their preferred body temperature [13]. When ambient temperatures are much higher (e.g. in deserts) or lower (e.g. at higher latitudes or altitudes) than the preferred core temperature, an endothermic animal has a higher field energy expenditure per mass unit than under ambient temperatures close to its core temperature. Diurnal or seasonal torpor, hibernation (throughout winter), and estivation (throughout summer) are states where individuals become relatively inactive and cease feeding to spare their food reserves [12]. Alternatively, migration to more thermally favourable habitats is a good option (e.g. birds in temperate and higher latitudes migrate to subtropical and tropical regions in the winter) when metabolic costs of endothermy become too high [13].

In extant ectotherms, the main source of internal heat in animals comes from the environment. Animals can thermoregulate behaviourally by exploiting different thermal microhabitats [12,13]. Basking in the sun or cooling in water is the most typical thermal behaviour seen in reptiles [14]. Winter torpor of reptiles is described as hibernation and is found in seasonal climates at moderate and high latitudes. In addition, many reptiles can, to some extent, adapt physiologically to changing temperatures [15]. Phenotypic changes in response to variation in environmental conditions (acclimatisation) can be facilitated by the number of mitochondria in cells [16], different metabolic isozymes [17-19], and regulation of transcription and expression of enzymes [20-22]. Migration to more favourable habitats is also an option for ectothermic animals to escape seasonal adverse environmental conditions [13].

Since the surface-to-volume ratio decreases with increasing body mass, the “inertial homeothermy hypothesis” under an ectothermic thermoregulation model has been suggested for large dinosaurs [1,2]. Large dinosaurs maintained higher, more constant body temperatures than smaller-sized reptiles, because large ectothermic animals heat up and cool down slower than smaller ectothermic animals (=gigantothermy). In other words, the body temperature of a dinosaur increases and body temperature fluctuations decrease with increasing body mass because of a decreasing surface-to-volume ratio with increasing body mass [3].

To test the inertial homeothermy hypothesis, Seebacher [3] developed a biophysical model that was calibrated with field data from eleven free-ranging crocodiles ( *Crocodylusporosus* [23]) and successfully validated on two other free-ranging crocodiles [24]. The body temperature of the crocodiles was measured with calibrated temperature-sensitive radio transmitters that animals of different masses swallowed and retained as pseudogastroliths in their stomachs. Body temperatures of the crocodiles were sampled during the whole day as well as during one summer and winter month to capture diurnal and seasonal variability. The biophysical model derived by Seebacher [3] predicted for crocodiles an increase in body temperature and decreasing fluctuations in body temperature with increasing body mass as expected under the inertial homeothermy hypothesis.

McNab [5] proposed a hypothesis on the limitation of dinosaurian metabolism and thus indirectly on the body temperature of dinosaurs, especially in large Theropoda and Sauropoda. The maximum size of vertebrates is determined by resource abundance and how it is used by a species. Assuming that the food intake of the largest herbivorous mammals defines the maximal rate at which terrestrial plant resources can be consumed, he demonstrated that the large size of sauropods is consistent with a field energy expenditure extrapolated from extant varanid lizards (corroborating Seebacher [3]). Analogously, assuming that the maximal size of carnivorous theropods is limited by the maximal capacity to consume vertebrates, as seen in extant terrestrial mammals, the size of the largest theropods agrees with a field energy expenditure extrapolated from varanid lizards (contrary to Seebacher [3]). From his calculations McNab [5] concluded that large herbivorous and carnivorous dinosaurs were homeothermic as a result of their very large body masses [25]. The dinosaurs in his model were not characterised by rates of metabolism seen in modern mammals and flighted birds, and had intermediate body temperatures. McNab [5] also noted a potential conflict with his model. Maximum growth rate estimates of large theropod and sauropod dinosaurs are large and close to those of modern mammals and precocial birds (scaled-up). The high growth rates could indicate a higher level of metabolism and thus higher body temperatures than observed in scaled-up varanid lizards. In amniotes (based on a dataset that includes *Varanusexanthematicus* and *Varanus* *niloticus* [26,27]; for ruminants [6]) a strong relationship between resting metabolic rate and growth rate has been shown.

Gillooly et al. [4] established a link between body temperature and maximum growth rate. In particular, they used an equation ( [28], hereafter MGR-T_b_-equation) to assess the average body temperature of animals T_b,MGR_ (°C), that is basically derived from the maximum growth rate, MGR (kg day^-1^) and the mass at maximum growth, M (kg) of the animal. This MGR-T_b_-equation relies on a ¾ power scaling of MGR with body mass. It additionally uses an Arrhenius approach to model body temperature effects on the biochemical reactions controlling individual growth and individual metabolic rate [29,30].

$$MGR=g_{0}\cdot M^{0.75}\cdot e^{\left(-E/k\cdot T\right)}$$ (1)

Rearranging the terms in equation (1) and setting Boltzmann’s factor *e* ^−*E*/*k*⋅*T*^ (E: average activation energy, k: Boltzmann’s constant, T: body temperature in Kelvin) to *e* ^0.1*T*^_*b*_ (T in °C) reveals the estimator T_b,MGR_ for body temperature (in °C) given in Gillooly et al. [4].

$$T_{b,MGR}=10\cdot \ln(MGR\cdot M^{-0.75}/g_{0})$$ (2)

Gillooly et al. [4] then estimated parameter g_0_ in equation (1) and the MGR-T_b_-equation (2) from data on scaling of maximum growth rates with body mass in reptiles [31] and in mammals [32]. Body temperature T_b_ was set to 30 °C for reptiles [33] and 37 °C for mammals [12]. This approach estimated parameter g_0_ as 1.7⋅10^−4^ (kg^1/4^ day^-1^) in reptiles and as 2.3⋅10^−4^ (kg^1/4^ day^-1^) in mammals. The estimation of g_0_ was based on the geometric mean of 12 estimates of *MGR*⋅*M* ^−0.75^ *e* ^0.1*T*^_*b*_ for reptiles [31] and on the mean of 163 estimates for mammals [32], respectively. Because g_0_ values of reptiles and mammals differed only slightly, Gillooly et al. [4] finally averaged the reptilian and mammalian g_0_ value (2⋅10^−4^ kg^1/4^ day^-1^) when applying their MGR-T_b_-equation to dinosaurs. Parameter values of MGR and of the asymptotic mass (M_A_) for dinosaurs were estimated from ontogenetic growth trajectories obtained from fossil long bones. Gillooly et al. [4] used trajectories of seven dinosaurs from a larger database of different dinosaurian lineages and geological periods to assess the body temperature of dinosaurs. The size of selected fully-grown dinosaurs ranged from 12 to 12,979 kg. Body temperature estimates of dinosaurs indicated a curvilinear increase in body temperature with the logarithm of body mass. While body temperatures of smaller dinosaurs were consistent with those seen in extant crocodiles (from the study of Seebacher et al. [23] and Seebacher [3]) and close to the average environmental temperature in their habitats (25 °C), the larger *Tyrannosaurus* *rex* and *Apatosaurus* *excelsus* had with approximately 33 °C and 41 °C, respectively clearly higher body temperatures than paleotemperature estimates (20-30 °C [3]) suggest. Gillooly et al. [4] concluded that dinosaurs were reptiles that exhibited inertial homeothermy. Since the observed relationship between body mass and body temperature was curvilinear and it predicted a body temperature for the largest dinosaurs (55,000 kg, 48°C) beyond the upper limit tolerated by most of today’s animals (45°C), Gillooly et al. [4] hypothesized that maximum body size in Dinosauria was ultimately limited by body temperature.

However, several more recent studies have questioned the results of Gillooly et al. [4]. First, the conclusion of Gillooly et al. [4] on the limitation of maximum size mathematically relies on the maximum growth rate estimate of the *Apatosaurus* specimen. This growth rate represents a clear overestimate [34-36]. Secondly, body temperatures calculated by Gillooly et al. [4] for dinosaurs contradict the ranges found in isotope thermometric studies [37,38].

In this paper, I analyse the accuracy of body temperature estimated from the MGR-T_b_-equation and revisit the hypothesis of Gillooly et al. [4] that the maximum body size in Dinosauria was ultimately limited by body temperature. First, I study whether body temperatures measured in today’s reptiles, birds and mammals are consistent with those predicted by the MGR-T_b_-equation I will therefore use datasets on core temperature of crocodiles [19,23], birds [39] and mammals [40] and compare these to respective body temperatures predicted from maximum growth rates. Second, I will apply the MGR-T_b_-equation to a larger data set of dinosaurs than those studied by Gillooly et al. [4] to study the relationship between body mass and body temperature in dinosaurs. This tests whether the results of Gillooly et al. [4] on inertial homeothermy and the limitation of maximal body size still hold for a larger number of dinosaurs. Finally, I will compare estimated body temperatures of dinosaurs to two models that have been suggested by other authors: a crocodile model [3] and a varanid lizard model [5].

## Materials and Methods

### Body temperatures in extant species and the MGR-T_b_-equation

The comparison of core temperatures (T_b_) measured in extant species and those calculated from the MGR-T_b_-equation (T_b,MGR_) was carried out for extant species from non-avian reptiles (Table S1), from precocial, and altricial birds (Table S2), as well as from marsupials and eutherian mammals (Table S3). For T_b_ of non-avian reptiles, I chose the field data on *Crocodylusporosus* from Seebacher et al. ( [23], N=10) and Seebacher [3] as well as from *Alligator* *mississippiensis* in Seebacher et al. ( [19], N=7). All reptilian T_b_s are annual averages obtained from calibrated temperature sensitive radio transmitters swallowed by the animals. T_b_s of mammals were extracted from the dataset of McNab ( [40], N=447) on basal metabolic rate and body temperature; for birds the dataset on T_b_ from McNab ( [39], N=88) was used. Since Case [32] has shown that scaling of MGR with body mass differs strongly between altricial and precocial bird species, I analysed the scaling of body temperature with mass in altricial and precocial birds separately. Bird species were assigned to a precocial or an altricial developmental mode following Dial [41]. Dial [41] distinguishes seven developmental stages of birds and assigns these to different bird orders. The precocial birds considered in my study (N=41), included all birds from McNab [39], belong to Dial’s [41] super-precocial, precocial or sub-precocial orders; the altricial birds (N=39) included those from Dials’s [41] semi-altricial, altricial and super-alticial orders. As the scaling of MGR with body mass differs between eutherian mammals and marsupials [32,42], the scaling of body temperature in these two mammalian lineages was also analysed separately (eutherian mammals: N=384; marsupials: N=63).

For the estimation of MGR from body mass, I used three different regressions for each taxon: one from Case ( [32]; hereafter Case-regression) and two from Werner and Griebeler [42]. The regressions from Werner and Griebeler [42] assume either that the slopes and intercepts are taxon-specific (hereafter MGR-regression) or that the slopes are fixed (0.75) and the intercepts are taxon-specific (as assumed in equation (1) and the MGR-T_b_-equation; hereafter fixed-slope-MGR-regression). The MGR-regression and the fixed-slope-MGR-regression linking log MGR to log body mass are based on much larger datasets on extant taxa than the respective regressions from Case [32]. Specifically for non-avian reptiles’ MGRs, three chelonians [43], five crocodiles (this study) and ten varanid lizards (this study) are added to the original dataset of Case [32] (N=66, Table S4). The fixed-slope-MGR-regression assumes an equal scaling of body temperature and MGR with body mass, resulting in an independence of T_b,MGR_ from body mass (equations 1 and 2). Thus, T_b,MGR_ values calculated from fixed-slope-MGR-regressions for a taxon can be interpreted as the average body temperatures in this taxon. If MGR scales with body mass at an exponent larger (smaller) than 0.75, body temperature estimated from the MGR-T_b_-equation increases (decreases) with increasing mass.

Since T_b,MGR_ is not only calculated from MGR but also from the mass at which MGR is observed, and there is a high natural variability in the body masses at maximum growth of species, I considered three different standard sigmoidal growth models to estimate the mass at maximum growth. These standard models had been successfully applied to ontogenetic growth series of non-avian reptiles, birds and mammals. Under the von Bertalanffy growth model ( [44,45], vBGM) MGR is found at about 30% (=100⋅8/27 [46]) of asymptotic mass (M_A_). In contrast, under the Gompertz growth model (GGM), MGR is about 37% (=100/*e* [46]), and under the logistic growth model (LGM) at 50% [46]. All three growth models have been successfully used to describe growth in extant non-avian reptilian taxa. The vBGM was used for extant snakes, lizards [47], turtles [48], crocodiles [49,50], and even extinct sauropod dinosaurs [34]. LGMs were applied to smaller extant reptiles [49] including tortoises [43] and to extinct dinosaurs from different lineages [36,51-54]. GGMs worked well for extant chelonians [31,55]. The increase in body mass of birds was successfully described by vBGMs [56], GGMs [57] and LGMs [58]. LGMs were applicable to extant eutherian mammals [59], but GGMs have also been used for mammals [59,60]. Based on these empirical observations, I considered for both non-avian reptiles and birds 30% of M_A_ (vBGM) as lower limit and 50% of M_A_ (LGM) as an upper limit of the body mass at maximum growth, and for mammals 37% of M_A_ (GGM) and 50% of M_A_ (LGM). My approach revealed an interval with T_b,MGR_ that is realistic for a species of a given body mass.

### Body temperatures in dinosaurs

#### Dinosaur specimen studied

Gillooly et al. [4] assessed body temperatures in dinosaurs based on the ontogenetic growth series of seven dinosaurs *Psittacosaurus* *mongoliensis* (12 kg), *Albertosaurus* *sarcophagus* (614 kg), *Gorgosaurus* *libratus* (622 kg), *Daspletosaurus* *torosus* (869 kg), *Tyrannosaurus* *rex* (2,780 kg), *Massospondylus* *carinatus* (140 kg), and *Apatosaurus* *excelsus* (12,979 kg) published in Erickson et al. [51,52]. Gillooly et al. [4] excluded based on the following arguments three specimens from these two papers: the feathered dinosaur bird *Shuvuuia* *deserti* (1.9 kg) with a presumed different thermoregulation than the other dinosaurs, *Syntarsusrhodesiensis* (18.8 kg) because the MGR of this species is an outlier, and *Maiasauruspeeblesorum* (1,660 kg) because of its bad growth curve (only three mass estimates). Hatchling weights predicted by the fitted growth curves of these three specimens are unrealistic ( *Shuvuuia* *deserti* : 0.45 kg compared to an asymptotic mass of 1.9 kg, *Syntarsusrhodesiensis* : 4.1 kg vs. 18.8 kg, *Maiasauruspeeblesorum* : 160 kg vs. 1,660 kg), providing further support for the exclusion of the three specimens from the study of Gillooly et al. [4]. I additionally excluded the growth curve of *D* *. torosus* from my analysis because it is only based on three mass estimates during ontogeny. I also excluded the curve of *A* *. excelsus* because the MGR of this specimen is clearly an overestimate [34-36]. In my analysis, I additionally considered more recently published growth curves of Archaeopteryx (0.9 kg) from Erickson et al. [53], of *Psittacosaurus* *lujiatunensis* (37.4 kg) from Erickson et al. [54], of *Alamosaurus* (32,000 kg) from Lehman and Woodward [34], of six sauropod dinosaur specimens (one mamenchisaurid sauropod (25,075 kg), two *Apatosaurus* sp. (18,178 kg, 20,206 kg), two indeterminate diplodocids (4,144 kg, 11,632 kg), and one *Camarasaurus* sp. (14,247 kg) from Griebeler et al. [36] and of one basal sauropodomorph dinosaur individual ( *Plateosaurus* *engelhardti* , 1,587 kg) from Griebeler et al. [36]. In total, for 15 dinosaurs belonging to five clades among Dinosauria (one *Archaeopteryx* individual, two Ceratosauroidea, four Tyrannosauroidea, two Prosauropoda and seven Sauropoda) I estimated body temperature from MGRs applying the MGR-T_b_-equation. For *Archaeopteryx* and *Plateosaurus* *engelhardti* the authors provided two and for *Alamosaurus* three growth models yielding different MGR estimates for each of these specimens, whereas for the other twelve dinosaurs only one growth curve is available. Overall, from 19 dinosaurian growth trajectories/ MGR estimates I estimated body temperatures (Table S5). Except for *Alamosaurus* (vBGM), LGMs had been successfully fitted by the authors to dinosaurs. To estimate T_b,MGR_ from the MGR-T_b_-equation, I therefore assumed for all dinosaurs that the mass at maximum growth is reached at half of the asymptotic mass, except for *Alamosaurus* (at 30%).

To test whether body temperature in dinosaurs (Sauropodomorpha, Sauropoda) increases with increasing body mass I established regressions linking estimated T_b,MGR_ from MGR and the mass at maximum growth to the logarithm of body mass of dinosaurs (Sauropodomorpha, Sauropoda) (M_A_). These regressions were calculated based on all dinosaurian MGRs (19), but also on all sauropodomorph MGRs (twelve) and sauropod MGRs (nine). From the results of Gillooly et al. [4] I expected the body temperature in dinosaurs (Sauropodomorpha, Sauropoda) to increase with increasing body mass.

I further studied T_b,MGR_ estimates of crocodiles and varanid lizards, because both taxa have been suggested as models for dinosaurs.

#### Crocodile model

Gillooly et al. [4] estimated body temperatures of crocodiles from the biophysical model developed by Seebacher [3] and considered a mean annual ambient temperature of 25 °C. Seebacher’s [3] biophysical model was calibrated with field data from eleven free-ranging crocodiles ( *Crocodylusporosus* ). However, the body temperatures from this field study are measurements of core temperatures of animals of different body mass. For dinosaurs, body temperature was estimated from the MGR-T_b_-equation and is thus based on growth in body mass under ambient temperature conditions. For this reason, I also calculated T_b,MGR_ from MGRs for crocodiles of different mass. To assess potential differences between T_b_ and T_b,MGR_, I additionally compiled literature for MGRs and adult body mass (M_A_) of crocodiles. The dataset of Case [32] comprises of only one data point for crocodiles ( *Alligator* *mississippiensis* ). For details on species, sources, methods, body masses of species, MGR estimates and calculated T_b,MGR_ please refer to (Table S6). When estimating T_b,MGR_ from the MGR-T_b_-equation for crocodiles, I assumed the mass at maximum growth as 30% of the body mass of the individual. Empirical studies have shown that growth in crocodiles follows a vBGM [49,50]. Finally, I established a regression line using all crocodilian data points (hereafter crocodile model) to test whether T_b_ [3], but also T_b,MGR_, increases with the logarithm of body mass. This would also test whether body temperatures estimated for dinosaurs fit to the crocodile model.

#### Varanid lizard model

McNab [5] had pointed out in his paper that the varanid lizards have 3.6 times higher rates of field energy expenditure than other lizards of equal size. As field energy expenditure is linked to metabolism [8], this could indicate higher body temperatures in varanid lizards than in other lizards and crocodiles of equal size. To the best of my knowledge, only one study on *Varanus* *varius* has measured core temperatures in varanid lizards [61] like Seebacher and colleagues [19,23] did for crocodiles. In this study, however, the varanid lizards were only monitored for 4 up to 13 days during summer, whereas Seebacher and colleagues monitored crocodiles over approximately one winter and summer month. Since intra-annual variability in environmental temperature was not captured in the study of *Varanus* *varius* a reliable comparison of T_b_ and T_b,MGR_ estimates was impossible for varanid lizards. Nevertheless, I was able to test whether body temperatures estimated for dinosaurs (T_b,MGR_) fit to this varanid lizard model. I therefore gathered information on MGRs and adult body mass (M_A_) of varanid lizards in literature. Note that no varanid lizard is included in the dataset of Case [32]. For details on species, sources, methods, body masses of species, MGR estimates and calculated T_b,MGR_ please refer to (Table S7). Since varanid lizards grow according to the vBGM [62,63], to estimate T_b,MGR_ from the MGR-T_b_-equation I assumed that the mass at maximum growth is 30% of the body mass of the individual. Based on the values of T_b,MGR_ and body mass of varanid lizards, I finally established a regression line (hereafter varanid lizard model) linking T_b,MGR_ to the logarithm in body mass in varanid lizards.

### Statistical analyses

In all extant taxa I analysed the relationships between body mass and T_b_ (T_b,MGR_) using ordinary linear least squares regression analysis. In dinosaurs the relationship between body mass and T_b,MGR_ was also analysed by ordinary least squares regression analysis, but I assumed both linear and non-linear models. In particular, I considered a non-linear model to test for a curvilinear increase in T_b,MGR_ with increasing body mass across all dinosaurs (Sauropodomorpha, Sauropodoa) that was expected from the results of Gillooly et al. [4]. In all regression analysis body mass was log-transformed, while T_b_ and T_b,MGR_ were not. The significance of differences in slopes and intercepts between two regression lines was tested by comparing the respective 95% confidence intervals of estimates. Overlapping confidence intervals of estimated slopes and intercepts indicate no statistical support (p > 0.05) of differences between regression lines. All statistical analyses were conducted in STATISTICA 7.1 (StatSoft, Inc. 1984-2005).

The estimation of T_b,MGR_ from individual MGR estimates and the MGR-T_b_-equation carried out for extant reptilian taxa and extinct dinosaurs was done in Excel 2003 (Microsoft Corporation).

## Results

### Body temperatures in extant species and the MGR-T_b_-equation

**Figure 1:**
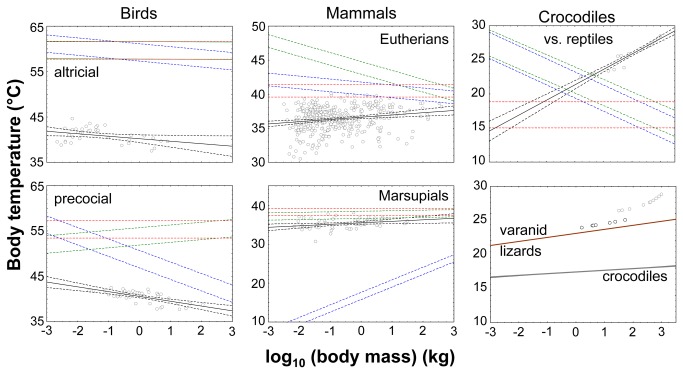
T_b_ and T_b,MGR_ against the logarithm of body mass in extant taxa. T_b_ in birds from McNab [39], in mammals from McNab [40] and in crocodiles from Seebacher [3], Seebacher et al. [19], and Seebacher et al. [23]. Bird species were assigned to a precocial or an altricial developmental mode following Dial [41]. For regressions linking T_b_ and T_b,MGR_, respectively to log body mass and statistics of regressions, please refer to Table 2. Black: regression line and 95% confidence interval of scaling of T_b_ in the taxon; blue: T_b,MGR_ derived from the Case-regression [32]; green: T_b,MGR_ derived from the MGR-regression [42]; red: T_b,MGR_ derived from the fixed-MGR-regression [42]; upper and lower limits of T_b,MGR_ were calculated based on different growth models that had been successfully applied to the taxon. Brown: my varanid lizard model (Table 2), grey: my crocodile model (Table 2).

Body temperatures predicted from the MGR-T_b_-equation (T_b,MGR_) did not fit very well to the T_b_ values for any of the three studied extant vertebrate lineages (Figure 1, Tables 1 and 2). This observation was independent of the three different regression functions used for estimating T_b,MGR_. Fixed-slope-MGR-regressions revealed, as expected, a constant body temperature for all studied vertebrate lineages that was independent of body mass, but differed strongly between lineages (Figure 1). T_b_ values predicted under the vBGM were always the highest. Those obtained from the GGM were intermediate, and those from the LGM revealed the lowest values for a given body mass (Figure 1).

**Table 1:** Logarithm of absolute maximum growth rate (g/day) against logarithm of body mass (kg) in extant taxa.

Taxon	Model	N	Slope	95% CI	Intercept	95% CI	R^2^	Source
Non-avian reptiles	Case-regression	42	0.666		-0.334			[32]
	MGR-regression	49	0.671		-0.288			[42]
	Fixed-MGR-regression	49	(0.75)		-0.273			[42]
Lacertilia	MGR-regression	18	0.634***	[0.329, 0.948]	- 0.323^n.s.^	[-0.905, 0.258]	0.545	
Serpentes	MGR-regression	15	0.701***	[0.457, 0.945]	-0.371*	[-0.689, -0.052]	0.748	
*Chelonia*	MGR-regression	10	0.603***	[0.337, 0.868]	- 0.205 ^n.s.^	[-0.698, 0.287]	0.694	
Crocodilia	MGR-regression	6	0.765 ^n.s.^	[-0.101, 1.630]	- 0.471 ^n.s.^	[-2.046, 1.103]	0.601	
Varanidae	MGR-regression	13	0.782***	[0.657, 0.908]	-0.162*	[-0.312, -0.012]	0.945	
Altricial birds	Case-regression	56	0.722		1.480			[32]
Altricial birds	MGR-regression	387	0.749		1.581			[42]
Altricial birds	Fixed-MGR-regression	387	(0.75)		1.583			[42]
Precocial birds	Case-regression	14	0.640		0.780			[32]
Precocial birds	MGR-regression	194	0.776		1.407			[42]
Precocial birds	Fixed-MGR-regression	194	(0.75)		1.396			[42]
Eutherian mammals	Case-regression	163	0.731		0.750			[32]
Eutherian mammals	MGR-regression	322	0.693		0.769			[42]
Eutherian mammals	Fixed-MGR-regression	322	(0.75)		0.794			[42]
Marsupials	Case-regression	4	0.820		-0.030			[32]
Marsupials	MGR-regression	21	0.756		-0.683			[42]
Marsupials	Fixed-MGR-regression	21	(0.75)		-0.697			[42]

Model: allometric regression used (for details refer to the text); slope, intercept: slope and intercept of the allometric regression; significance levels: n.s. p > 0.05, *p ≤ 0.05, **p < 0.01, ***p < 0.001; 95% CI: 95% confidence interval; R^2^: variance explained by the linear regression.

**Table 2:** Body temperature (° C) against the logarithm of body mass (kg) in extant taxa.

Taxon	Body temperature	Scaling model	M_inflection point_	Slope	Intercept
Non-avian reptiles	T_b,MGR_	Case	vBGM	1.934	23.236
	T_b,MGR_	MGR	vBGM	1.819	23.950
	T_b,MGR_	Fixed-MGR	vBGM	(0.75)	18.838
Non-avian reptiles	T_b,MGR_	Case	LGM	1.934	19.405
	T_b,MGR_	MGR	LGM	1.819	20.119
	T_b,MGR_	Fixed-MGR	LGM	(0.75)	15.007
Crocodilia	T_b,MGR_		vBGM	0.341	14.270
Crocodilia	T_b_			2.263***	21.331***
Varanidae	T_b,MGR_		vBGM	0.744	21.396
Altricial birds	T_b,MGR_	Case	vBGM	0.645	61.137
	T_b,MGR_	MGR	vBGM	0.023	61.600
	T_b,MGR_	Fixed-MGR	vBGM	(0.75)	61.574
Altricial birds	T_b,MGR_	Case	LGM	0.645	57.305
	T_b,MGR_	MGR	LGM	0.023	57.766
	T_b,MGR_	Fixed-MGR	LGM	(0.75)	57.743
Altricial birds	T_b_			-0.548*	40.217***
Precocial birds	T_b,MGR_	Case	vBGM	-2.533	50.683
	T_b,MGR_	MGR	vBGM	0.599	55.726
	T_b,MGR_	Fixed-MGR	vBGM	(0.75)	57.268
Precocial birds	T_b,MGR_	Case	LGM	-2.533	46.852
	T_b,MGR_	MGR	LGM	0.599	51.894
	T_b,MGR_	Fixed-MGR	LGM	(0.75)	53.437
Precocial birds	T_b_			-1.058*	40.574***
Eutherian mammals	T_b,MGR_	Case	GGM	0.437	41.784
	T_b,MGR_	MGR	GGM	1.312	44.801
	T_b,MGR_	Fixed-MGR	GGM	(0.75)	41.439
Eutherian mammals	T_b,MGR_	Case	LGM	0.437	39.921
	T_b,MGR_	MGR	LGM	1.312	42.937
	T_b,MGR_	Fixed-MGR	LGM	(0.75)	39.576
Eutherian mammals	T_b_			0.329***	36.622***
Marsupials	T_b,MGR_	Case	GGM	3.224	17.630
	T_b,MGR_	MGR	GGM	0.138	36.605
	T_b,MGR_	Fixed-MGR	GGM	(0.75)	39.205
Marsupials	T_b,MGR_	Case	LGM	3.224	15.767
	T_b,MGR_	MGR	LGM	0.138	36.605
	T_b,MGR_	Fixed-MGR	LGM	(0.75)	37.342
Marsupials	T_b_			0.385*	35.492***

Comparison of T_b,MGR_ and T_b_. T_b,MGR_ was estimated from different allometric regressions linking the log of maximum growth rate (MGR) to the log of body mass (Case-regression, MGR-regression, and fixed-MGR-regression; for details refer to the text and Table 1). M_inflection point_: mass at the maximum growth rate of the individual used in the MGR-T_b_-equation, vBGM (30% of asymptotic mass of the individual), GGM (37%) and LGM (50%). T_b_: body temperatures of vertebrate taxa from different datasets [3,19,23,39,40]. Slope, intercept: slope and intercept of the linear regression linking body temperature to log body mass. Significance levels: n.s. p > 0.05, *p ≤ 0.05, **p < 0.01, ***p < 0.001.

#### Non-avian reptiles

As expected [3], T_b_ in crocodiles significantly increased with increasing body mass (Table 2). In contrast, when applying the Case-regression or the MGR-regression to extant non-avian reptiles, T_b,MGR_ decreased with increasing body mass. T_b,MGR_ values derived from the fixed-slope-MGR-regression on non-avian-reptiles (vBGM: 18.838 °C; LGM: 15,007 °C) were on average considerably lower than the T_b_ values of crocodiles (mean 26.635 ± standard deviation s.d. 2.175 °C).

**Figure 2:**
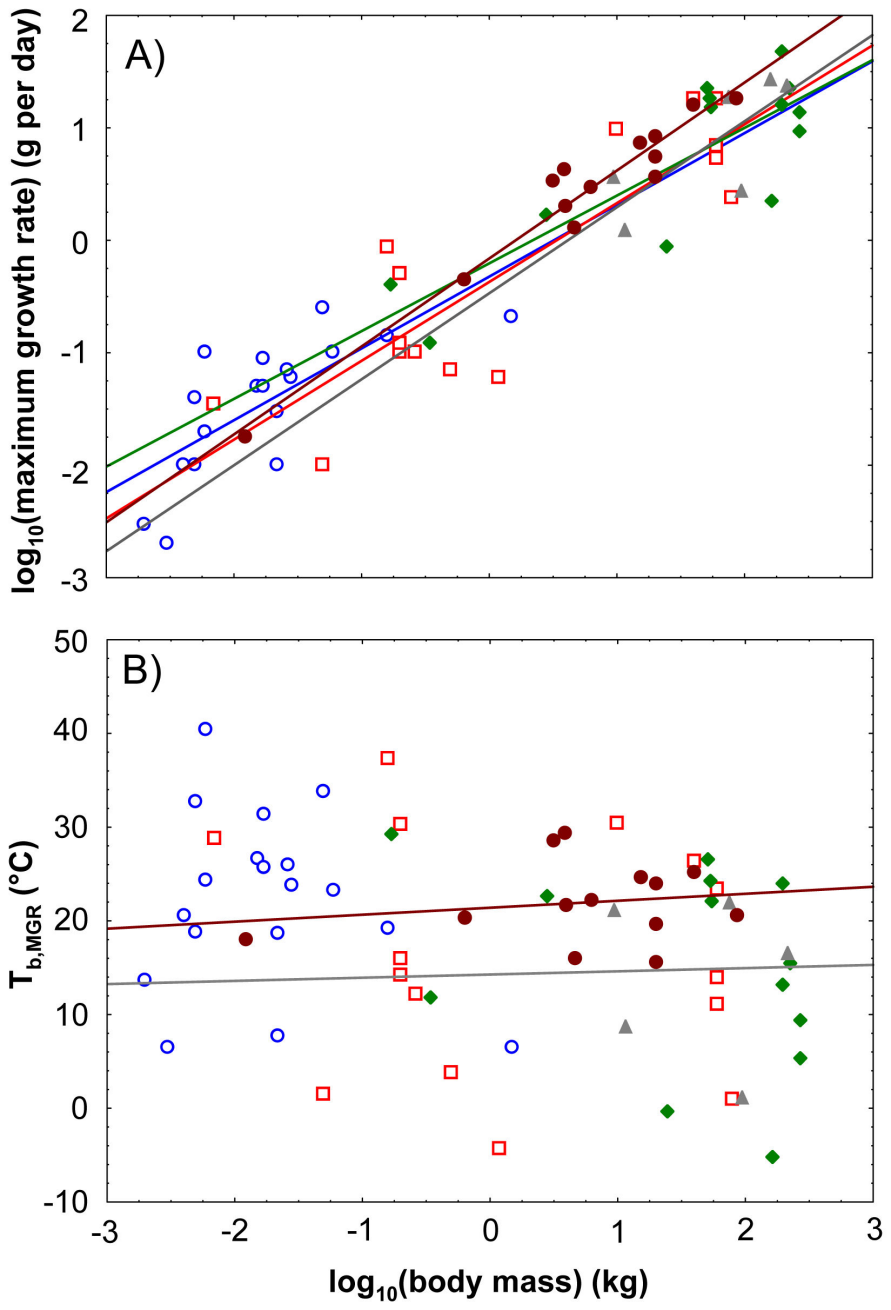
MGR and T_b,MGR_ against the logarithm of body mass in extant non-avian reptiles. Log MGR is shown in panel (A) and T_b,MGR_ in (B). For regressions on log MGR and T_b,MGR_, respectively against log body mass, please refer to Tables 1 and 2. Lacertilia (open blue dots, blue line), Serpentes (open red squares, red line), *Chelonia* (filled green diamonds, green line), Crocodilia (grey filled triangles, grey line) and Varanidae (filled brown dots, brown line).

Different scaling regression lines linking MGR to log body mass were derived for Lacertilia, Serpentes, *Chelonia* , Crocodilia and Varanidae (Table 1, Figure 2), but none of the slopes and intercepts differed significantly between these taxa.

#### Precocial, and altricial birds

T_b_ in precocial and altricial birds significantly decreased with increasing body mass (Table 2). On average, T_b_ in precocial birds (mean 40.520 ± s.d. 1.328 °C) was slightly lower than in altricial birds (mean 40.969 ± s.d. 1.654 °C), but this difference was not significant. T_b,MGR_ in precocial and altricical birds based on the Case-regression and the MGR-regression also decreased with increasing body mass. T_b,MGR_ values estimated from the respective Case-regression and MGR-regression for precocial and altricial birds were unrealistically higher than the respective T_b_ values (Figure 1). T_b,MGR_ values derived from the respective Case-regression and MGR-regression for altricial birds exceeded those of precocial birds. T_b,MGR_ estimated from the fixed-slope-MGR-regression of precocial birds was 53.427 °C under the vBGM and 57.268 °C under the LGM and for altricial birds 57.743 °C and 61.674 °C, respectively. Thus all T_b,MGR_ of birds were clearly physiologically unrealistic.

#### Marsupials and eutherian mammals

T_b_ in marsupials and eutherian mammals significantly increased with increasing log body mass (Table 2). Marsupials had on average (mean 35.275 ± s.d. 1.296 °C) a lower T_b_ than eutherian mammals (mean 36.365 ± s.d. 1.752 °C). T_b,MGR_ values estimated from the Case-regression and MGR-regression for marsupials increased again with increasing body mass, whereas T_b,MGR_ of eutherian mammals decreased for both regressions. T_b,MGR_ estimated from the fixed-slope-MGR-regression of marsupials was 39.205 °C under the GGM, and 37.342 °C under the LGM; for eutherian mammals 41.439 °C, and 39.576 °C, respectively. Thus, T_b,MGR_ values of marsupials and eutherian mammals showed the lowest deviation from the respective T_b_ within the three studied extant vertebrate lineages.

### Body temperatures in dinosaurs

**Figure 3:**
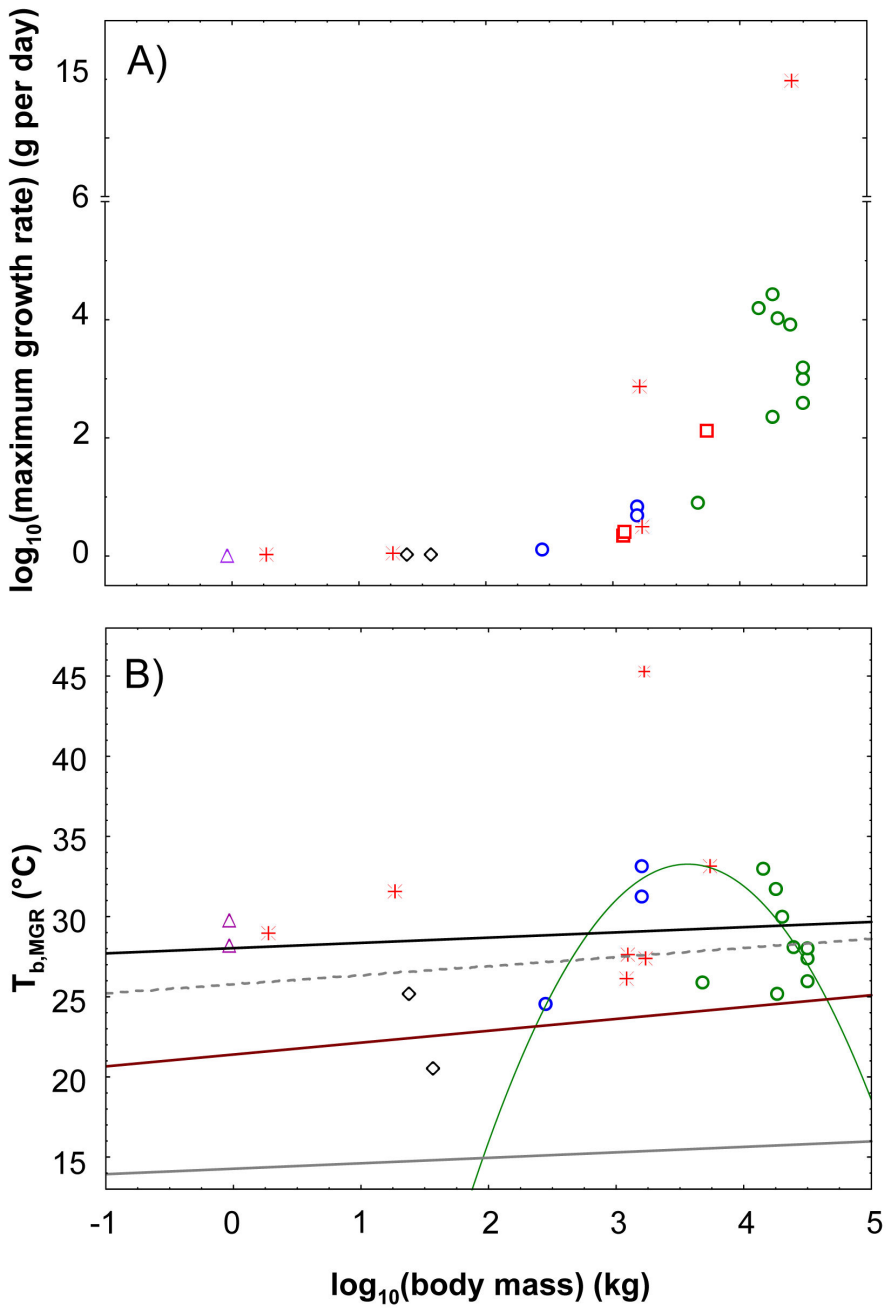
MGR and T_b,MGR_ against the logarithm of body mass in dinosaurs. Log MGR is shown in panel (A) and T_b,MGR_ in (B). Open symbols (included in this study [34,36,51-54]): sauropods (green dots), prosauropods (blue dots), theropods (red squares), ornithischians *Psittacosaurus* (black diamond), *Archaeopteryx* (purple triangle); red crosses (excluded from the study Erickson et al.[51,52].): *Shuvuuia* *deserti*, *Syntarsus* *rhodesiensis*, *Maiasaurus* *peeblesorum*, *Daspletosaurus* *torosus*, *Apatosaurus* *excelsus*; black line: overall scaling of T_b,MGR_ in dinosaurs, green line: curvature of T_b,MGR_ in Sauropodomorpha (MfN.R.2625 from Griebeler et al. [36] excluded, Table 3); grey solid line: my crocodile model, grey dashed line: crocodile model from Gillooly et al. [4]; brown line: my varanid lizard model. For statistics of regressions please refer to Tables 2 and 3.

T_b,MGR_ was independent of body mass (linear scaling, slope: p > 0.05, Table 3, Figure 3) across all dinosaurs (28.033 °C), all Sauropodomorpha (28.712 °C) and all Sauropoda (28.712 °C). In Sauropodomorpha (Table 3, Figure 3), however, a curvilinear (quadratic polynomial) relationship between body temperature and body mass was significant after excluding the indeterminate diplodocid (MfN.R.2625) from the dataset. The MGR estimate of this specimen is the poorest of the seven Sauropodomorpha studied in Griebeler et al. [36]. In comparison to the other Sauropodomorpha studied in this paper the MfN.R.2625 specimen has the lowest number of growth cycles preserved (9 vs. 9-22) and its growth record does only document the linear phase of growth, which hampers a good fit of a sigmoidal growth model [36]. Three other Sauropodomorpha specimens having also nine growth cycles preserved were not excluded ( *Camarasurus* sp. from Griebeler et al. [36], *Alamosaurus* *sanjuanensis* from Lehman and Woodward [34], *Massospondylus* *carinatus* from Erickson et al. [52]) because their growth records clearly document a sigmoidal growth trajectory.

**Table 3:** Scaling of T_b,MGR_ (° C) with the logarithm of body mass (kg) in dinosaurs.

Taxon	Model	N	β_0_	95% CI	β_1_	95% CI	β_2_	95% CI	R^2^
all dinosaurs	linear	19	26.460***	[22.481, 30.439]	0.520^n.s.^	[-0.624, 1.664]			0.051
Sauropodomorpha	linear	12	40.261***	[24.247, 56.275]	- 2.750^n.s.^	[-6.640, 1.140]			0.221
Sauropodomorpha	quadratic	12	- 27.061^n.s.^	[-92.306, 38.183]	32.406^n.s.^	[-5.037, 69.850]	- 4.514n.s.	[-9.712, 0.684]	0.221
Sauropodomorpha without MfN. R.2625	quadratic	11	-56.863*	[-110.116, -3.610]	50.617**	[19.686, 81.548]	-7.106**	[-11.422, -2.790]	0.364
Sauropoda	linear	9	27.890^n.s.^	[-11.793, 67.577]	0.099^n.s.^	[-9.145, 9.342]			0.001
Sauropoda without MfN. R.2625	linear	8	21.634^n.s.^	[-12.506, 55.772]	1.418^n.s.^	[-6.502, 9.338]			0.031
Prosauropoda	linear	3	- 0.226^n.s.^	[-80.712, 80.260]	10.124^n.s.^	[-16.989, 37.219]			0.958
Theropoda	linear	3	- 2.928^n.s.^	[-87.315, 81.560]	9.628^n.s.^	[-15.760, 35.015]			0.959

Model: linear T_b,MGR_ = β_0_ + β_1_ M, quadratic T_b,MGR_ = β_0_ + β_1_ M + β_1_ M^2^; significance levels: n.s. p > 0.05, *p ≤ 0.05, **p < 0.01, ***p < 0.001; 95% CI: 95% confidence interval; R^2^: variance explained by the regression model.

Except for *Psittacosaurus* *lujiatunensis* , T_b,MGR_ of all dinosaurs studied were higher than predicted by the varanid lizard model. As the varanid lizard model revealed higher T_b,MGR_ values for dinosaurs than the crocodile model, T_b,MGR_ were also higher than under the crocodile model (Figure 3).

## Discussion

### Body temperatures in extant species and the MGR-T_b_-equation

The overall dependency (increase, decrease, independence) between T_b_ and log body mass was correctly reproduced by the regressions linking T_b,MGR_ to log body mass in crocodiles, birds and marsupials, but not in eutherian mammals. In crocodiles, both T_b,MGR_ (derived from the MGR-regression) and T_b_ increased with increasing body mass. This positive scaling of body temperature is consistent with the results of Seebacher [3] and corroborates the inertial homoeothermy for crocodiles not only for T_b_, but also for T_b,MGR_.

In precocial birds, altricial birds and marsupials, both T_b,MGR_ (derived from the Case-regression and the MGR-regression) and T_b_ significantly decreased with increasing body mass. In contrast, in eutherian mammals T_b_ significantly increased and T_b,MGR_ values (derived from the Case-regression and the MGR-regression) decreased with increasing body mass.

Most of my results on the dependencies between T_b_ and log body mass in extant species are corroborated by other studies. Based on an analysis of a very small data set on birds and mammals, Rodbard [64] argued that T_b_ inversely scales with body mass in both lineages. McNab [39] was able to corroborate his finding using a larger dataset for birds, but demonstrated different scaling in T_b_ for different taxonomic groups within mammals. White and Seymour [65] compiled an extensive dataset on mammals and found an overall increase in T_b_ with increasing body mass, which is contrary to Rodbard [64]. The most recent extensive study on scaling of T_b_ in mammals and birds is the one of Clarke and Rothery [66]. Contrary to all other studies before, these authors examined the variation in T_b_ associated without and with phylogeny. When ignoring phylogenetic effects (as I did) their analysis supported the results of McNab [39], a positive scaling of T_b_ in mammals and an inverse scaling in birds. When allowing for phylogenetic effects in their analysis, the inverse scaling in birds was corroborated but no relationship between body mass and T_b_ in mammals was identified. Within taxonomic groups of birds and mammals, a positive scaling, a negative scaling and no relationship between T_b_ and body mass was observed by Clarke and Rothery [66]. Contrary to the negative scaling found in my study for altricial and precocial birds, Clarke and Rothery [66] showed a weakly positive scaling of T_b_ in the altricial Passeriformes [41]. In the altricial Piciformes and precocial Anseriformes [41], T_b_ was independent of body mass. Differences in scaling relationships between taxonomic groups were even more pronounced in mammals than in birds and differed between orders [66]. Contrary to my results, the scaling of T_b_ was positive in marsupials, but this overall relationship was not statistically supported for any marsupilian order [66]. The results of Clarke and Rothery [66] recommend that any overall relationship between T_b_ and body mass in a taxon should be interpreted cautiously because the overall pattern of scaling is strongly influenced by the mixture of different scaling relationships existing at lower phylogenetic levels and their proportion of species in the sample. Nevertheless, for both birds and eutherian mammals Clarke and Rothery [66] observed that in taxonomic groups containing species of a large body size, scaling of T_b_ is negative. In non-passerine birds, artiodactyles and carnivores big species have a lower T_b_ than smaller species. This negative scaling of T_b_ in larger birds and eutherian mammals is corroborated by T_b,MGR_, and suggest that the MGR-T_b_-equation is useful to assess in larger species of mammals and birds whether body temperature is independent of log body mass or scales positive or negative.

However, for a given body mass/ species the accuracy of T_b,MGR_ in comparison to T_b_ was low and strongly differed between the vertebrate lineages studied. For endothermic birds and mammals, body temperatures predicted by the MGR-T_b_-equation (T_b,MGR_) for a species of a given body mass were always higher than T_b_; for ectothermic crocodiles, T_b_ was much higher than T_b,MGR_. Nevertheless, the ranking seen in T_b_ values of extant taxa was well reflected in T_b,MGR_. Altricial birds have the highest T_b_ and T_b,MGR_ values, and both are lower than in precocial birds. T_b_ and T_b,MGR_ values in eutherians are lower than in birds, and crocodiles have the lowest T_b_ and T_b,MGR_.

**Figure 4:**
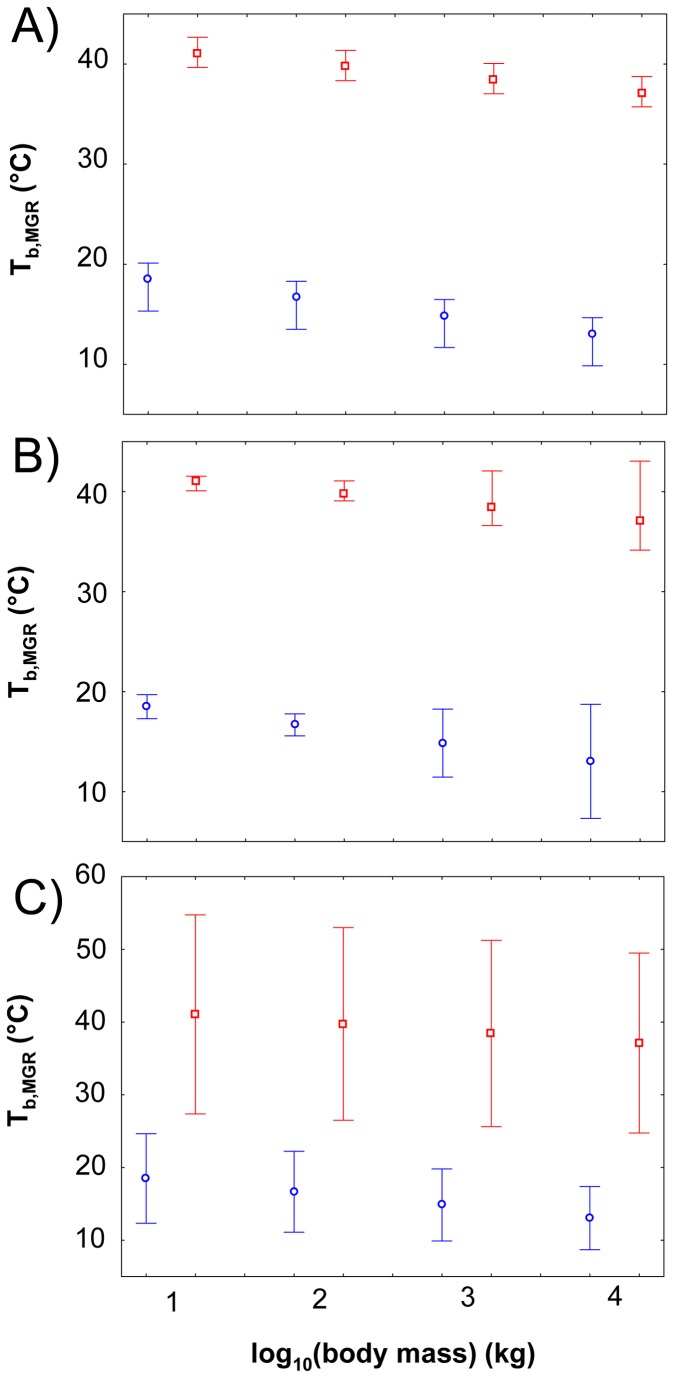
Results of the sensitivity analysis on the influence of values assumed for g_0_, the scaling exponent of MGR, and the activation energy term *e* ^0.1T^_*b*_ on estimated T_b,MGR_ of extant (non-avian) reptilian and mammalian species. For different body masses (1, 10, 100, 1000 kg), T_b,MGR_ was calculated from the MGR-T_b_-equation in Gillooly et al. [4] applying the MGR-regression for reptiles (blue) and mammals (red), respectively to estimate MGR from body mass (Table 1). Tested parameter values: (A) in the MGR-T_b_-equation g_0_ was set to 0.00017 (reptiles [4]), 0.0002 (average of reptiles and mammals [4]) and 0.00023 (mammals [4]); (B) scaling exponent used in the MGR-T_b_-equation was 0.65, 0.75 (default) and 0.85; (C) 0.075, 0.1 and 0.15 was used as an exponent in the activation energy term*e* ^0.1*T*^_*b*_, or an activation energy of 0.447, 0.65 and 0.894 eV, respectively. The average values used by Gillooly et al. [4] result in average T_b,MGR_ for reptiles (open dots) and mammals (open squares). The reptilian g_0_ (upper whisker mark) reveals higher T_b,MGR_ than the mammalian value (lower whisker mark). Scaling exponents smaller than 0.75 (upper whisker mark) result in higher T_b,MGR_ and higher exponents (lower whisker mark) in lower T_b,MGR_ than observed under a ¾ scaling of MGR. Note: MGR scales in non-avian reptiles with about 0.65, in mammals and birds with about 0.75 (Table 1). An exponent of 0.075 in the activation energy term (upper whisker mark) reveals the highest T_b,MGR_ and an exponent of 0.15 the lowest T_b,MGR_ (lower whisker mark). Note: Average activation energies of non-avian reptiles (0.757 eV), mammals (0.856 eV) and birds (1.005 eV) are all higher than the 0.65 eV used in the MGR-T_b_-equation [67]. A usage of the specific activation energies for these three vertebrate lineages results in lower T_b,MGR_ values than predicted by the MGR-T_b_-equation. The average activation energy of ectothermic fish is 0.433 eV (Downs et al. 2008 [67], upper whiskers).

Several hypotheses could explain the quantitative differences between T_b_ and T_b,MGR_, which are considerably larger in birds and crocodiles than in mammals. First, the MGR-T_b_-equation (T_b,MGR_) was calibrated by Gillooly et al. [4] to reveal T_b,MGR_ values of 30 °C for reptiles and 37 °C for mammals. These values were identified with g_0_ = 2⋅10^−4^kg^1/4^ day^-1^ thereby assuming a ¾ scaling of MGR (equation 1) and an average activation energy of 0.65 eV (term*e* ^0.1*T*^_*b*_, equation 1) for the biochemical reactions underlying the metabolism of an individual. However, the specific g_0_ estimated by Gillooly et al. [4] for reptiles was 1.7⋅10^−4^ kg^1/4^ day^-1^ and for mammals 2.3⋅10^−4^ kg^1/4^ day^-1^. The value of g_0_ of reptiles was based only on twelve species, whereas g_0_ of mammals was based on 163 species. The ¾ scaling of MGR underlying the MGR-T_b_-equation is not observed in all vertebrate taxa, although for none of the taxa studied herein a deviation from a ¾ scaling is statistically significant ( [42], Table 1). Downs et al. [67] have shown that also the activation energy differs between taxonomic groups. While in birds (1.005 ± 0.212 eV) and in mammals (0.856 ± 0.068 eV) the activation energy is on average much higher than assumed by the MGR-T_b_-equation (0.65 eV), in reptiles the activation energy (0.757 ± 0.043 eV) is closer to this value. Nevertheless, according to a translation of activation energy in Q_10_ values, the taxon-specific activation energies of birds, mammals and reptiles still correspond to the typical range of Q_10_ for whole body metabolism (i.e. Q_10_ c. 2-3 over the range of 0-40°C [67]). Figure 4 displays the results of my small sensitivity analyses. The analysis was carried out to gain insights into the influence of the values assumed for g_0_, for the scaling exponent of MGR and for the activation energy on estimated T_b,MGR_ for species of different body masses. Errors in T_b,MGR_ introduced by averaging g_0_ of non-avian reptiles and mammals are very small. Setting g_0_ for reptiles to 1.7⋅10^−4^ kg^1/4^ day^-1^ and for mammals to 2.3⋅10^−4^ kg^1/4^ day^-1^ (instead of 2⋅10^−4^ kg^1/4^ day^-1^ as assumed by the MGR-T_b_-equation) increased T_b,MGR_ by about 2°C in reptiles and decreased T_b,MGR_ by about 2°C in mammals. Errors introduced by a deviation from a ¾ scaling of MGR increase with increasing body mass. Smaller exponents than 0.75 (0.65, reptiles, Table 1) lead to higher T_b,MGR_ and higher exponents (0.85, marsupials, Table 1) to lower T_b,MGR_ for reptiles and mammals for the body masses studied in my sensitivity analysis. Whereas for a 1 kg reptile or mammal the error introduced by a deviation of the exponent from 0.75 is low (about 2°C), for a reptile or mammal with a body mass of 1,000 kg it is already about 6°C (0.65 scaling exponent, Table 1). Small errors in the activation energy resulted in even stronger changes in T_b,MGR_ as predicted by the MGR-T_b_-equation. Specifically, for reptiles, mammals and birds, all having on average larger activation energies than 0.65 eV [67], T_b,MGR_ considerably decreased when the correct activation energy was used in the MGR-T_b_-equation. For example, an activation energy of 0.89 eV (mammals) decreases T_b,MGR_ of a mammal between 7 and 8 °C. For crocodiles, an average (non-avian) reptilian activation energy (0.757 eV [67]) results in an even stronger underestimation of T_b_ [19,23] by T_b,MGR_. In total, my small sensitivity analyses suggests that values assumed in the MGR-T_b_-equation for g_0_, the scaling exponent of MGR and the average activation energy can introduce very large inaccuracies in estimated body temperatures of species (Figure 4).

For reptiles I found a strong underestimation of T_b_ by T_b,MGR_. A known caveat of the MGR-T_b_-equation [68] linking individual growth to body mass (M_A_) is the disregard for reproduction in West et al. [28] and Gillooly et al. [4]. For the so-called determinate growers (most mammals and birds) modelled by the MGR-T_b_-equation, all growth occurs before reproduction begins. In so-called indeterminate growers (many fish and non-avian reptiles), individuals continue to grow after first reproduction. Thus, in indeterminate growers growth is substantially slowed down before reaching M_A_ because materials and energy are not only allocated to individual growth and maintenance but also to reproduction. A lower MGR results in lower body temperatures predicted by the MGR-T_b_-equation. This inherent underestimation of T_b_ in indeterminate growers by the MGR-T_b_-equation is important in extant non-avian reptiles, but could also be significant in several dinosaurs presumed to reproduce well before reaching full size M_A_ [35,36,69]. In non-avian reptiles, growth can also be highly variable, reflecting environmental inconsistencies within and between years in general [70] and in ambient temperatures in particular [70,71]. For example, the most northerly distributed extant crocodilian species, the American Alligator, stops eating when ambient temperature drops below 16 °C. It is only during the warmer months of the year during active feeding that growth occurs [71]. During winter torpor (hibernation), growth in non-avian reptiles stops completely [70]. Since MGR of larger reptiles and dinosaurs (annual growth marks are preserved in long bones; for a review on the establishment of growth trajectories, see 35,36) is calculated at a yearly basis, phases of growth and not growth within the year are averaged. Annual MGRs (although transformed to a daily basis) therefore underestimate the real maximum daily growth rate of the specimen. For example, if an American alligator with a body mass of 160 kg and a MGR of 27.0 g per day estimated at a yearly base [32] does not grow between October and March (= 6 months [71]), the respective (daily) MGR is doubled when only referring to the growth phase (54 g per day) and T_b,MGR_ rises from 20.02 to 26.95 °C. This revised T_b,MGR_ is very close to a T_b_ of 26.24 °C (= 25 + 1.24 °C, with 25 °C average annual temperature [4]) estimated from the biophysical model of Seebacher [3] and to the average T_b_ of about 24 °C measured by Seebacher et al. [19] in a field study on the American alligator (Figure 1). In conclusion, the MGR-T_b_-equation underestimates T_b_ for non-avian reptiles when a species shows considerable, long phases of no growth within the year. This underestimation could explain the higher ranges of body temperatures found in isotope thermometric studies for dinosaurs [37,38] than by Gillooly et al. [4].

The accuracy of estimated T_b,MGR_ was best in mammals which is expected because Gillooly et al. [4] calibrated the MGR-T_b_-equation based on this vertebrates. For eutherian mammals T_b,MGR_ values derived from the fixed-slope-MGR-regression and the Case-regression were closer to T_b_ than the T_b,MGR_ values derived from MGR-regression. In particular, the MGR-regression revealed unrealistically high T_b_ values for animals smaller than 1 kg (Figure 1). However, differences in the slope of the MGR-regression and the Case-regression are not significant and they include the 0.75 of the fixed-slope-MGR-regression [42]. Thus, the higher T_b,MGR_ derived from the MGR-regression compared to the other two regressions (0.731 for Case-regression and 0.75 for fixed-slope regression, Table 1) are not statistically supported. The generally higher T_b,MGR_ values derived from the fixed-slope-MGR-regression and the Case-regression are consistent with a higher activation energy observed in mammals (0.856 ± 0.068 eV [67]) than assumed by the MGR-T_b_-equation (0.65 eV) (Figure 4).

For marsupials T_b,MGR_ values derived from the MGR-regression and the fixed-slope regression were close to T_b_ values. Contrarily, the Case-regression revealed unrealistically low T_b,MGR_ values for marsupials, but this regression is only based on four species (Table 1). In marsupials a ¾ scaling of MGR assumed in the MGR-T_b_-equation is indeed observed ( [42], Table 1). Thus a lower g_0_ and/or higher activation energy than assumed by the MGR-T_b_-equation could have caused the small overestimation of T_b,MGR_ by the MGR-regression and the fixed-slope-MGR-regression in marsupials.

However, for birds, I found the strongest overestimation of T_b_ by the MGR-T_b_-equation. This is contrary to the other determinate growers, mammals. While body temperatures of adult birds and mammals are very similar, differences in metabolic rates exist between these two taxa attributed to the expensive and expansive form of avian flight. White et al. [72] found that smaller (< 1 kg) birds have a higher standard metabolic rate (normalized to 38 °C) than mammals (about 1.2 times at a mass of 10 g), whereas in larger birds the opposite is true. Based on a very extensive analysis, McNab [40,73] suggested that birds have on average basal metabolic rates 30-40% greater than mammals. Since both studies demonstrated only small differences in the metabolism of adult bird and mammal individuals, these results are unable to fully explain the large differences seen between T_b_ and T_b,MGR_ in birds over a body mass range of five orders of magnitude. However, the observation that birds generally have higher T_b_ and T_b,MGR_ than mammals is consistent with the results of Western and Ssemakula [74]. Western and Ssemakula [74] found that most of the variation in MGR observed between birds and mammals can be attributed to body temperature, metabolic rate and brain weight (e.g. primates have very large brains compared to other species of equal size and grow slower).

Altricial birds and precocial birds have MGRs about five times and three times higher than eutherian mammals (fixed-MGR-regression, Table 1), but these values are reached in this determinate growers during the juvenile phase. In altricial nestlings, the thermoregulation and muscle coordination develops slowly during the growth phase and parents heat the young by sitting on the nest. In contrast, the young of precocial birds are endothermic and quite mobile after hatching [75]. The resulting energy saved in altricial young compared to precocial young during the juvenile phase could at least partially explain the higher MGRs in altricial than in precocial birds. Case [32] formulated a preliminary idea explaining the large difference in MGR of precocial and altricial birds. Birds which grow quickly are fed frequently by both parents, while slow growers are either self-feeding or are fed large food parcels at infrequent intervals by their parents. In many altricial birds, e.g. passerines, growth rates are very high; the lowest avian growth rates have been measured in the young of precocial and self-feeding birds. Ricklefs [58] confirmed in a model his alternative hypothesis, that interspecific variation in growth rates of altricial birds is the result of adaptations to levels of predation and the requirement for, and availability of, energy to the nestling. This model questions the reasoning of Case [32]. Independent of factors driving the differences in MGR between altricial and precocial young, altricial chicks save energy during the juvenile phase compared to precocial chicks. This energy could be allocated to their growth.

In conclusion, my results on the comparison of T_b_ and T_b,MGR_ in different extant vertebrate lineages suggest that the dependency (increase, decrease, independence) between body mass and body temperature can be assessed from the MGR-T_b_-equation for crocodiles, birds, and larger mammals. However, the accuracy of T_b,MGR_ derived from this equation was poor in all vertebrate lineages studied. Taxon-specific differences in the scaling of MGR (g_0_, scaling exponent) and in the activation energy of biochemical reactions assumed in Arrhenius model as well as ecological and evolutionary adaptations of species cause the observed differences in T_b_ and T_b,MGR_. This suggests that we cannot expect that the MGR-T_b_-equation will reveal accurate body temperatures for dinosaurs. This in turn strongly questions the applicability of the MGR-T_b_-equation to study a potential limitation of body mass in Dinosauria due to overheating.

### Body temperatures in dinosaurs

Irrespective of the inaccuracy of T_b,MGR_ values observed in extant species I expected a curvilinear increase of T_b,MGR_ with increasing log body mass in dinosaurs from the results of Gillooly et al. [4]. But contrary to my expectation, across all dinosaurs, Sauropodomorpha and Sauropoda, T_b,MGR_ was independent of body mass (linear scaling of T_b,MGR_ with increasing log body mass, Table 3). All T_b,MGR_ values derived for dinosaurs were largely consistent with paleotemperature estimates (20-30 °C [3]). These two results strongly contradict Gillooly et al. [4] and also question the conclusion of these authors on the limitation of body mass in Dinosauria. Only *Plateosaurus* , *Apatosaurus* (BYU601-17328) and *Tyrannosaurus* *rex* had slightly higher T_b,MGR_ than 30°C [3]. The overall range of T_b,MGR_ of dinosaurs (24.55-31.12 °C; *Massospondylus* *carinatus* , *T. rex*) was smaller than the range of T_b,MGR_ seen in extant non-avian reptiles (-5.29-40.47 °C; *Caretta* *caretta* , *Cnemidophorus* *sexlineatus* ), extant crocodiles (1.04-21.89 °C; *Crocodylusporosus* , female American alligator) and extant varanid lizards (15.57-29.33 °C; *Varanussalvator* , *Varanus* *niloticus* ). The lower variability of T_b,MGR_ found at a given body size in the larger Dinosauria compared with the smaller variability seen in extant non-avian reptiles conforms with inertial homeothermy in Dinosauria. According to the biophysical model of Seebacher [3], larger ectothermic animals have more stable body temperatures than smaller. However, reasons for the natural variability seen in growth rates of extant similar-sized individuals are not only ambient temperature and thermoregulation but also food availability, quality and intake, and water availability [70]. Moreover, the sample size of studied extant non-avian reptiles is much larger than that of Dinosauria. We can expect that the natural variability covered by a smaller sample is lower than by a larger sample, even if two (statistical) populations have equal ranges.

While in the two prosauropods T_b,MGR_ increases with increasing body mass, in sauropods T_b,MGR_ decreases with increasing body mass. However, neither the trend in T_b,MGR_ of prosauropods nor the trend in T_b,MGR_ of sauropods is statistically significant. Nevertheless, a curvilinear relationship between T_b,MGR_ and body mass was significant when excluding the MfN.R.2625 specimen from the dataset (Figure 3, Table 3). The decrease in body temperature with increasing body mass in sauropods, which is statistically supported by the fitted parabola (Figure 3), again strongly contradicts the hypothesis that the body mass of the largest dinosaurs was ultimately limited by body temperature. This is not to say that sauropods did not exhibit inertial homeothermy [3,5], but that they were able to efficiently cool themselves down [76].

For all dinosaurs studied, T_b,MGR_ values predicted by my crocodile model were lower than the T_b,MGR_ values derived from the varanid lizard model. The higher T_b,MGR_ of varanid lizards compared to crocodiles supports McNab [5]. The aggressively predatory varanid lizards have considerably higher field energy expenditures and metabolic rates than most other lizards [5].

Except for *Psittacosaurus* , in all dinosaurs studied T_b,MGR_ values were even higher than assumed under my varanid lizard model. This model was inspired by the energetics model developed by McNab [5] that illustrates the link between food intake and metabolic rate. When assuming that the food intake of the largest herbivorous (carnivorous) mammals defines the maximal rate at which a terrestrial environments’ plant resources (vertebrate species) can be consumed, McNab [5] showed that the large size of sauropods (carnivorous theropods) is consistent with a field energy expenditure extrapolated from extant ectothermic varanid lizards. This shows a significantly lower metabolic rate in sauropods and theropods than in extant endothermic mammals and birds. Since body temperature is linked to metabolic rate, the high T_b,MGR_ (compared to extant varanid lizards) of all dinosaurs studied is not in accordance with food intake under an ectothermic metabolism of extant varanid lizard. This could indicate a higher rate of metabolism in dinosaurs than in varanid lizards (as already pointed out in McNab [5]. The observation that T_b,MGR_ is more or less consistent with paleotemperature estimates (20-30 °C [3]) in all dinosaurs studied could eventually question endothermy in these dinosaurs. The latter argument against endothermy in dinosaurs, however, is based on precise estimates of T_b_ in dinosaurs, which are unfortunately not derivable from the MGR-T_b_-equation.

In total, the high inaccuracy of dinosaurian T_b,MGR_ as evidenced by the application of the MGR-T_b_-equation to different extant vertebrate lineages makes a reliable test of the limitation of maximal body size in Dinosauria impossible. Irrespective of this inaccuracy of body temperatures a larger dataset of dinosaurian MGRs than studied by Gillooly et al. [4] provided no support for this hypothesis.

## Supporting Information

Table S1 **Body mass and body temperature of crocodiles studied.**
(XLS)

Table S2 **Body mass and body temperature in birds.**
(XLS)

Table S3 **Body mass and body temperature in mammals.**
(XLS)

Table S4 **Body mass, maximum growth rate and source of data of reptiles studied.**
(XLS)

Table S5 **Body mass, maximum growth rate and source of data of dinosaurs studied.**
(XLS)

Table S6 **Sex, adult mass, estimated maximum daily growth rates and sources of data for several species of Crocodilians.**
(DOC)

Table S7 **Neonate mass, sex, adult mass, estimated maximum daily growth rates and sources of data for several species of the genus *Varanus.***
(DOC)
