# Supplementary material for: Body Temperatures in Dinosaurs: What Can Growth Curves Tell Us?
Source: PLoS One. 2013 Oct 30;8(10):e74317. doi: 10.1371/journal.pone.0074317 (PMC3812988; doi:10.1371/journal.pone.0074317)
Supplement: Table S6 — Sex, adult mass, estimated maximum daily growth rates and sources of data for several species of Crocodilians. (DOC) [file pone.0074317.s006.doc]

**Establishment of the MGR-regression for Crocodilians**

I compiled data on crocodilian growth rates and adult body masses in the literature (Table S2). For all analysed species information on growth was given for snout-vent length (*L*). I transformed *L* to mass (*M*), using an allometric equation of the form *M* = *aL^b^* (Andrews 1982). Parameters *a* and *b* were roughly estimated from the ten total length-mass pairs of *Crocodylus porosus* given in Seebacher et al. (1999). For *Crocodylus porosus*, the American alligator, and *Caiman crocodilus crocodiles* the respective study directly provided an estimate on maximum growth rate. For *Caiman latirostris* a von Bertalanffy growth function was given in the paper. I finally plotted adult body mass vs. maximum growth rate per day (log-log-plot) and fitted a regression line to the data (Figure 1 and 2).

**Table S2. Sex, adult mass, estimated maximum daily growth rates and sources of data for several species of Crocodilians*.***

| Species | Sex | Adult mass (g) | Estimated MGR  (g per day) | Sources |
| --- | --- | --- | --- | --- |
| *Crocodylus. porosus* | population average | 95623 | 2.75 | Sah & Stuebing (1996) |
| *Alligator mississippiensis* | male | 214712 | 23.67 | Wilkinson & Rhodes (1997) |
|  | female | 76337 | 18.68 | Wilkinson & Rhodes (1997) |
|  | unknown | 160000 | 27.00 | Case (1978) |
| *Caiman crocodilus crocodilus* | population average | 9551 | 3.62 | Magnusson & Sanaiotti (1995) |
| *Caiman latiostris* | population average | 11602 | 1.21 | Moulton et al. (1999) |

**References (Crocodilians)**

Seebacher F, Grigg GC & Beard LA (1999) Crocodiles as dinosaurs: behavioural thermoregulation in very large ectotherms leads to high and stable body temperatures. *The Journal of Experimental Biology* 202: 77-86

Sah AMS & Stuebing RB (1996) Diet, growth and movement of juvenile crocodiles *Crocodylus porosus* Schneider in the Klias River, Sabah, Malaysia. *Journal of Tropical Ecology* 12: 651-662

Wilkinson PM & Rhodes WE (1997) Growth rates of American alligators in costal South Carolina. *The Journal of Wildlife Management* 61:397-402

Case TJ (1978) On the evolution and adaptive significance of postnatal growth rates in the terrestrial vertebrates. *The Quarterly Review of Biology* 53: 243-282.

Magnusson WE & Sanaiotti TM (1995) Growth of *Caiman crocodilus crocodilus* in Central Amazonia, Brazil. *Copeia* 2: 498-501

Moulton TP, Magnusson WE & Melo MTQ (1999) Growth of *Caiman latirostris* inhabiting a costal environment in Brazil. *Journal of Herpetology* 33: 479-484
