# Supplementary material for: Body Temperatures in Dinosaurs: What Can Growth Curves Tell Us?
Source: PLoS One. 2013 Oct 30;8(10):e74317. doi: 10.1371/journal.pone.0074317 (PMC3812988; doi:10.1371/journal.pone.0074317)
Supplement: Table S7 — Neonate mass, sex, adult mass, estimated maximum daily growth rates and sources of data for several species of the genus Varanus. (DOC) [file pone.0074317.s007.doc]

**Establishment of the MGR-regression for Varanidae**

I gathered data on growth rates of neonate varanid lizards and their adult body mass increase from literature (Table S1). For *V. salvator* and *V. varius*, I found a time series for ontogenetic increase in body mass. In the taxa for which only growth curves for snout-vent length (*L*) were available, I transformed those to mass (*M*), using an allometric equation of the form *M* = *aL^b^* (Andrews 1982). Parameters *a* and *b* were either estimated from time series for snout-vent length and respective *M* (*Varanus niloticus*) or from the length-mass pairs [(neonate length, neonate mass) and (adult length, adult mass)] obtained from the literature sources (*V. brevicauda*, *V. griseus*). For *V. komodoensis*, the first study directly provided an estimate of maximum growth rate and the second study contained information on the average daily gain in body mass of several individuals. I then plotted adult body mass vs. maximum growth rate per day (log-log-plot) and fitted a regression line to the data (Figure 1 and 2).

**Table S1. Neonate mass, sex, adult mass, estimated maximum daily growth rates and sources of data for several species of the genus *Varanus.***

| Species | Neonate mass (g) | Sex | Adult mass (g) | Estimated MGR  (g per day) | Sources |
| --- | --- | --- | --- | --- | --- |
| *V. brevicauda* | 1.1 | unknown | 12.3 | 0.018 | James (1996) |
| *V. griseus* | 19.3 | unknown | 647.0 | 0.447 | Thompson & Pianka (2001)  Pianka et al. (2004)  Smirina & Tsellarius (1996) |
| *V. varius* | 1.3 | unknown | 4700.0 | 1.281 | Boylan (1995) |
| *V. niloticus* | 28.3 | male | 15171.1 | 7.279 | Buffrénil & Héméry (2002) |
|  | 31.5 | female | 6271.4 | 2.969 | Buffrénil & Héméry (2002) |
|  | 25.2 | male | 6250.0 | 2.905 | Buffrénil & Héméry (2002) |
|  | 40.5 | female | 3945.8 | 1.975 | Buffrénil & Héméry (2002) |
|  | 36.7 | male | 3926.7 | 4.249 | Buffrénil & Héméry (2002) |
|  | 34.8 | female | 3188.6 | 3.348 | Buffrénil & Héméry (2002) |
| *V. salvator* | 35.0 | female | >20000.0 | 3.636 | Andrews & Gaulke (1990),  Jessop *et al.* (2006)  Andrews & Gaulke (1990) |
|  | 50.0 | female | >20000.0 | 5.455 | Andrews & Gaulke (1990)  Jessop *et al.* (2006)  Andrews & Gaulke (1990) |
|  | 48.0 | male | >20000.0 | 8.354 | Andrews & Gaulke (1990)  Andrews & Gaulke (1990) |
| *V. komodoensis* | 95.2 | unknown | 40000.0 | 16.000 | Jessop *et al.* (2006)  Nowak (1999) |
|  | 95.2 | unknown | 87000.0 | 18.000 | Jessop *et al.* (2006)  Lemm et al. (2004)  Lemm et al. (2004) |

**References (Varanidae)**

Andrews RM (1982) Patterns of growth in reptiles. *Biology of the Reptilia 13, Physiology D*, eds Gans C & Pough HF (John Wiley and Sons, New York), pp 273-320.

James CD (1996) Ecology of the pygmy goanna (*Varanus brevicauda*) in spinifex grasslands of central Australia. *Australian Journal of Zoology* 44:177-192.

Thompson GG & Pianka ER (2001) Allometry of clutch and neonate sizes in monitor lizards (Varanidae: *Varanus*). *Copeia* 2001(2):445-458.

Pianka ER, King DR, & King RA (2004) *Varanoid lizards of the world* (Indiana University Press, Bloomington) p 608.

Smirina EM & Tsellarius AY (1996) Aging, longevity, and growth of the desert monitor lizard (*Varanus griseus* Daud.). *Russian Journal of Herpetology* 3(2):130-142.

Boylan T (1995) Field observations, captive breeding and growth rates of the lace monitor, *Varanus varius*. *Herpetofauna* 25(1):10-14.

Buffrénil Vd & Héméry G (2002) Variation in longevity, growth, and morphology in exploited nile monitors (*Varanus niloticus*) from Sahelian Africa. *Journal of Herpetology* 36(6):419-426.

Andrews HV & Gaulke M (1990) Observations on the reproductive biology and growth of the water monitor (*Varanus salvator*) at Madras crocodile bank. *Hamadyrad* 15(1):1-5.

Jessop TS*, et al.* (2006) Maximum body size among insular Komodo dragon populations covaries with large prey density. *Oikos* 112:422-429.

Nowak RM (1999) Walker’s Mammals of the World (Johns Hopkins University Press, Baltimore).

Lemm JM, Edwards MS, Grant TD, & Alberts AC (2004) Comparison of growth and nutritional status of juvenile Komodo monitors (*Varanus komodoensis*) maintained on rodent or poultry-based diets. *Zoo Biology* 23:239-252.
